# Beta cell dysfunction induced by bone morphogenetic protein (BMP)-2 is associated with histone modifications and decreased NeuroD1 chromatin binding

**DOI:** 10.1038/s41419-023-05906-w

**Published:** 2023-07-05

**Authors:** Adriana Ibarra Urizar, Michala Prause, Lars Roed Ingerslev, Matthew Wortham, Yinghui Sui, Maike Sander, Kristine Williams, Romain Barrès, Martin R. Larsen, Gitte Lund Christensen, Nils Billestrup

**Affiliations:** 1 Department of Biomedical Science, University of Copenhagen, Copenhagen, Denmark; 2 Novo Nordisk Foundation Center for Basic Metabolic Research, University of Copenhagen, Copenhagen, Denmark; 3 Departments of Pediatrics and Cellular & Molecular Medicine, Pediatric Diabetes Research Center, University of California, San Diego, La Jolla, CA 92093 USA; 4 Institut de Pharmacologie Moléculaire et Cellulaire, CNRS and Université de Nice Côte d’Azur, Valbonne, France; 5 Department of Biochemistry and Molecular Biology, University of Southern Denmark, Odense, Denmark

**Keywords:** Cell division, Type 2 diabetes, Epigenetics, Transcriptomics, Protein-protein interaction networks

## Abstract

Insufficient insulin secretion is a hallmark of type 2 diabetes and has been attributed to beta cell identity loss characterized by decreased expression of several key beta cell genes. The pro-inflammatory factor BMP-2 is upregulated in islets of Langerhans from individuals with diabetes and acts as an inhibitor of beta cell function and proliferation. Exposure to BMP-2 induces expression of *Id1-4*, *Hes-1*, and *Hey-1* which are transcriptional regulators associated with loss of differentiation. The aim of this study was to investigate the mechanism by which BMP-2 induces beta cell dysfunction and loss of cell maturity. Mouse islets exposed to BMP-2 for 10 days showed impaired glucose-stimulated insulin secretion and beta cell proliferation. BMP-2-induced beta cell dysfunction was associated with decreased expression of cell maturity and proliferation markers specific to the beta cell such as *Ins1*, *Ucn3*, and *Ki67* and increased expression of *Id1-4*, *Hes-1*, and *Hey-1*. The top 30 most regulated proteins significantly correlated with corresponding mRNA expression. BMP-2-induced gene expression changes were associated with a predominant reduction in acetylation of H3K27 and a decrease in NeuroD1 chromatin binding activity. These results show that BMP-2 induces loss of beta cell maturity and suggest that remodeling of H3K27ac and decreased NeuroD1 DNA binding activity participate in the effect of BMP-2 on beta cell dysfunction.

## Introduction

Type 2 diabetes mellitus (T2D) is characterized by relative insulin deficiency caused by insulin resistance and beta cell dysfunction. The observed progressive deterioration of beta cell mass and function in T2D has traditionally been associated with apoptosis [1, 2]. However, the dedifferentiation of beta cells towards less mature and dysfunctional cells has been suggested as an alternative explanation for the loss of insulin-positive cell mass in T2D [3]. In mouse models of T2D, beta cell dedifferentiation and loss of beta cell identity have been associated with decreased expression of several transcription factors that are crucial for the maintenance of beta cell function, including FoxO1 [3], Nkx6.1 [4] and NeuroD1 [5, 6]. This has also been described in some studies of human islets [6, 7], although there is considerable heterogeneity of T2D islet datasets, with the extent of beta cell identity marker repression being dependent on methodology and patient cohort [8, 9]. Moreover, hyperglycemia, inflammation, and oxidative stress are all factors associated with T2D, and are thought to alter the expression of these beta cell-specific transcription factors [3, 10–12] and thus contribute to beta cell dedifferentiation.

Bone morphogenetic proteins (BMPs) are members of the TGF-β family of growth and differentiation factors and are known to play central roles in pancreas and islet development [13, 14]. Moreover, emerging data points to BMPs, particularly BMP-2 and -4, as inflammatory factors, which are also associated with obesity, T2D, and beta cell dysfunction [15–19]. mRNA expression of *BMP-2* and -*4* is upregulated in pancreatic islets of *db*/*db* mice, and *BMP-2* expression is induced by pro-inflammatory cytokines in rodent and human islets in vitro [18, 20, 21]. In human islets, the expression of *BMP-2* is correlated positively with donor HbA1c, suggesting a potential role in beta cell dysfunction [22, 23]. Further, BMP-2 and -4 have been described to act as potent inhibitors of beta cell proliferation and function in vitro in both rodent and human islets [18, 24].

BMP-2 and -4 bind to the BMP type I receptor, activin-like kinase (ALK)-3 and -6, and forms a complex with the BMP type II receptors. This receptor complex stimulates the activation of SMAD1/5/8 and subsequently the expression of inhibitory basic helix-loop-helix (bHLH) factors, such as the four Inhibitor of DNA binding proteins (Id1-4), Hairy and enhancer of split-1 (Hes-1), and Hairy/enhancer-of-split related with YRPW motif protein 1 (Hey-1) [25, 26]. The Id proteins as well as Hey-1 and Hes-1, are known to inhibit the transcriptional activity of other central beta cell bHLH factors, including NeuroD1, [25, 27, 28] and are known to inhibit differentiated cell function in many cells types [29, 30]. Specifically in beta cells, both Id and Hes-1 expression are associated with poor beta cell function and dedifferentiation [31–33].

Epigenetic modifications play a central role in cellular differentiation and maintenance of cellular identity and disruption of epigenetic modifications can have pathophysiological consequences [34]. Histone modifications have been shown to be involved in beta cell differentiation, maintenance of identity, functional adaptation, and proliferation [35–39]. Moreover, evidence suggests that epigenetic mechanisms may be involved in the pathogenesis of diabetes [38, 40–43]. Polycomb-dependent histone methylation events have been implicated in beta cell dedifferentiation in both mouse and human beta cell dysfunction resulting in increased expression of genes associated with immature beta cell phenotype and a decrease in expression of genes associated with mature beta cells [38]. BMPs induce chromatin remodeling in several tissues such as osteoblasts and fibroblasts by recruiting or inducing the expression histone modifying enzymes [44, 45]. Furthermore, Hes-1 and Hey-1 recruit histone deacetylases [46, 47]. Thus, BMPs are likely to induce epigenetic changes that regulate cell differentiation either directly through SMADs or through their downstream targets such as the Id’s, Hes-1 and Hey-1.

It has been suggested that loss of beta cell identity occurs in islets of patients with T2D. However, the molecular mechanism of this reprogramming to less mature beta cells has not been elucidated in detail. In the present study, we investigate the role of BMP-2 in this process by applying a multi-omics approach (transcriptome, proteome, and epigenome) aimed at elucidating BMP-2 effects. BMP-2 is produced locally by islets when exposed to inflammation [21] and increased BMP-2 serum concentrations have been found in T2D patients [19, 23]. We hypothesize that prolonged exposure of pancreatic islets to BMP-2 results in epigenetic alterations affecting the expression of key beta cell genes leading to loss of beta cell maturity and dysfunction.

## Results

### BMP-2 induces beta cell dysfunction and inhibition of proliferation

**Fig. 1:**
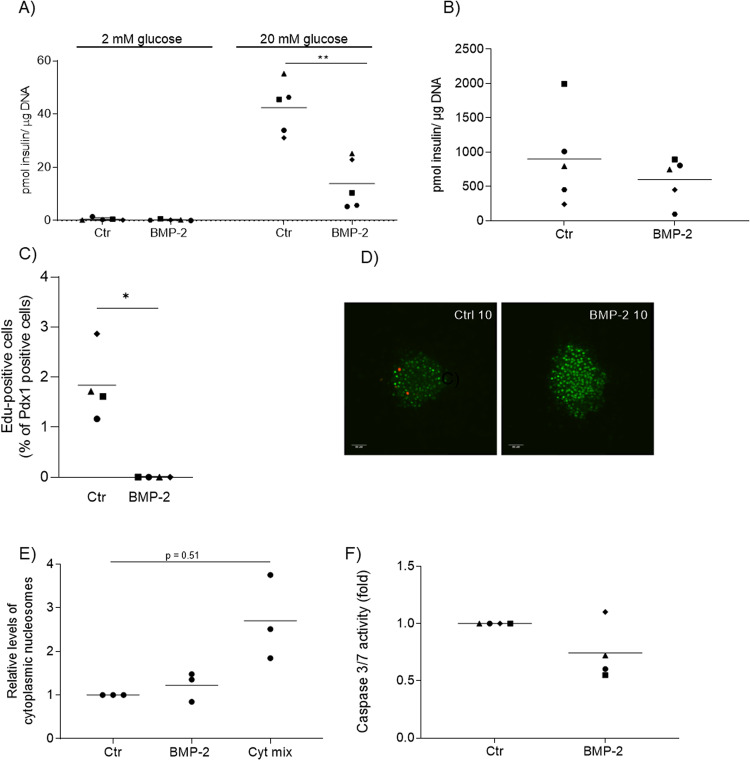
BMP-2 induced beta cell dysfunction and inhibition of proliferation. Mouse islets were exposed to BMP-2 (50 ng/ml) for 10 days or left non-exposed. **A** Insulin secretion was measured by static batch incubations for 30 min in response to 2 mmol/l glucose followed by 20 mmol/l and normalized to DNA content. Data were shown as means *N* = 5, **p < 0.01 two-sided paired *t*-test vs. ctr. **B** Total insulin content was measured post-GSIS. Data were shown as means for *N* = 5, two-sided t-test vs ctr. **C** Beta cell proliferation was examined in whole mouse islets exposed to BMP-2 for 10 days. Proliferation was determined by immunocytochemical staining for Pdx-1 and Click-iT detection of EdU. Results are shown as the percentage of proliferating Pdx-1 positive cells. Data were shown as means for *N* = 4, *p < 0.05, two-sided paired *t*-test vs. ctr. **D** Immunocytochemical and Click-iT staining of whole mouse islets. Cells were stained for Pdx-1 (green) and EdU (red). The data shown is representative. **E** Apoptosis was measured by cytoplasmic nucleosome levels in lysates from mouse islets exposed as described above. Two-day stimulation with a cytokine mix of IL-1β (300 pg/ml) and IFN-γ (10 ng/ml) served as a positive control for the induction of apoptosis. Data were shown as means for *N* = 3, two-sided paired *t*-test vs ctr. **F** Caspase 3/7 activity was measured in lysates from mouse islets exposed as described above. Data were shown as means for *N* = 4.

In order to study the effects of long-term BMP-2 exposure on beta cell function and proliferation, primary mouse islets were isolated and cultured in vitro for 10 days in the presence or absence of BMP-2 (50 ng/mL). Exposure of islets to BMP-2 for 10 days resulted in a significant reduction of glucose-stimulated insulin secretion (GSIS) (Fig. 1A) without significantly affecting total islet insulin content (Fig. 1B).

Next, we examined whether BMP-2 affected beta cell proliferation in cultures of intact mouse islets. Proliferating beta cells were identified by EdU incorporation into Pdx-1 positive cells. After 10 days, control islets showed 1.8% Pdx-1 positive cells whereas no beta cell proliferation was observed after BMP-2 exposure (Fig. 1C, D). To determine whether the inhibition of GSIS and proliferation by BMP-2 was associated with apoptosis, we analyzed islet cell apoptosis. Exposure of islets to BMP-2 for 10 days did not induce islet cell apoptosis measured as the release of cytoplasmic nucleosomes (Fig. 1E) nor caspase 3/7 activation (Fig. 1F).

### BMP-2 induces changes in markers of beta cell maturity

We hypothesize that BMP-2-induced beta cell dysfunction is associated with changes in the expression of beta cell maturity genes and genes involved in cell proliferation. Total RNA was isolated from islets exposed to BMP-2 for 10 days or left non-exposed and selected beta cell-specific gene expressions were analyzed by qRT-PCR.

**Fig. 2:**
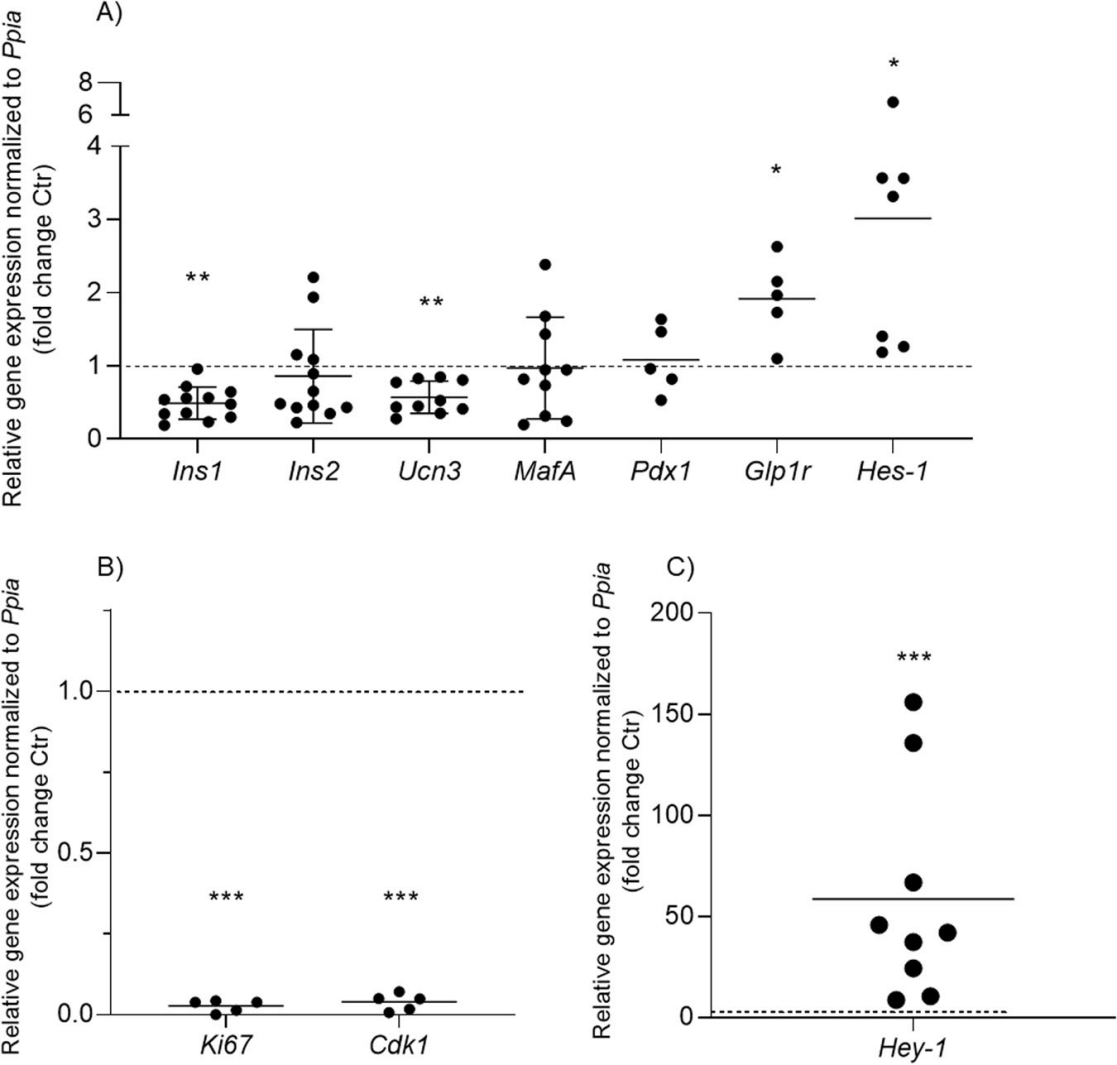
BMP-2 induced changes of key beta cell gene expression. Mouse islets were exposed to BMP-2 (50 ng/ml) or left non-exposed for 10 days. Relative mRNA expression of **A** *Ins1*, *Ins2*, *Ucn3, MafA, Pdx1, Glp1r*, and *Hes-1*, *N* = 5–12, **B** *Ki67* and *Cdk1*, *N* = 5, and **C** *Hey-1*, *N* = 9 was analyzed using real-time PCR. Expression levels are normalized to control *Ppia* and Ctr data were set to 1 represented by the dotted horizontal line. Data were shown as means ± SD, *p < 0.05; **p < 0.01, ***p < 0.001 2-sided paired *t*-test vs. ctr on log10 transformed data.

BMP-2 significantly reduced expression of *Ins1* and *Ucn3* mRNAs in mouse islets compared to control islets (Fig. 2A). No significant differences were observed in mRNA expression of *Ins2*, *MafA*, or *Pdx1*, whereas *Glp1r* expression was significantly upregulated ~2-fold by BMP-2 exposure. The proliferation markers *Ki67* and *Cdk1* were significantly reduced by BMP-2 to nearly non-detectable levels (Fig. 2B) supporting the observation of BMP-2-inhibited beta cell proliferation.

The expression of known BMP-2 target genes was also analyzed. BMP-2 exposure for 10 days significantly induced *Hes-1* expression by ~3.5-fold (Fig. 2A) and *Hey-1* mRNA expression by ~60-fold in primary mouse islets (Fig. 2C).

**Fig. 3:**
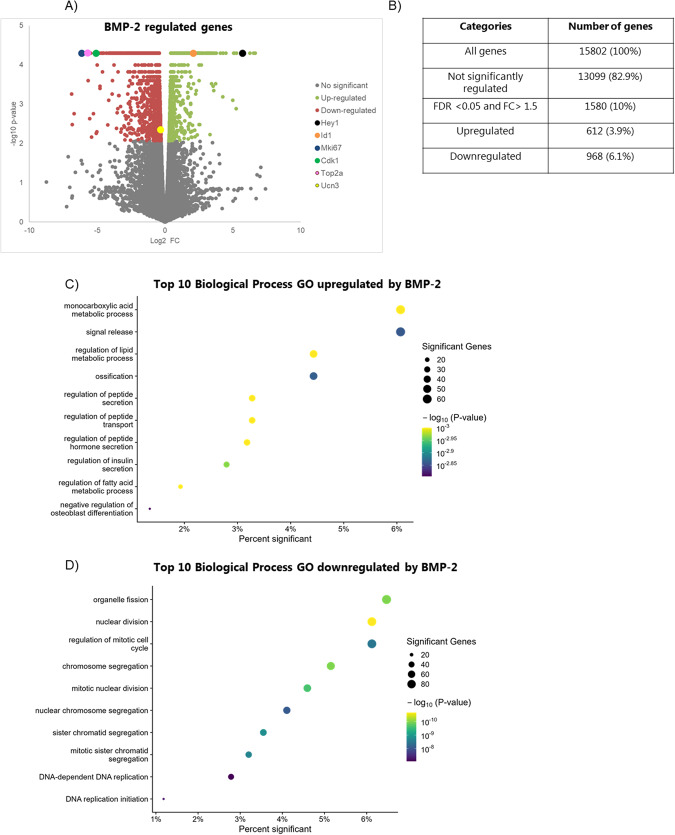
BMP-2 exposure affects global RNA expression. Mouse islets were exposed to BMP-2 (50 pg/ml) or left non-exposed for 10 days. **A** Volcano plot representing the differential expressed RNA’s between BMP-2 vs. ctr exposed mouse islets. X-axis: log2 gene expression fold-change, Y-axis: −log10 *p* value. Each dot represents an individual RNA. Gray: not significantly regulated genes, green: FDR <0.05 upregulated genes, and red FDR <0.05 downregulated genes. Selected regulated genes by BMP-2 are marked. **B** Table showing the summary of genes regulated by BMP-2. **C**, **D** Top ten most significantly enriched biological processes GO of BMP-2 upregulated and downregulated genes, respectively. X-axis: Percent of genes in the GO significantly regulated by BMP-2. Circle size: Number of significant BMP-2 regulated genes in the GO.

The transition toward a less mature and dysfunctional beta cell requires changes in the transcription factor network regulating multiple genes. To characterize the transcriptional effects of BMP-2 exposure more globally, we performed mRNA-sequencing (mRNA-seq). Comparison of gene expression profiles of mouse islets exposed to BMP-2 or left non-exposed showed significant differences in the expression of 1580 genes (FDR <0.05 and FC >1.5), of which 612 were upregulated and 968 were downregulated by BMP-2 (Fig. 3A, B).

Consistent with the qRT-PCR data, the expression of *Ucn3* was significantly reduced by BMP-2 as was the expression of the proliferation markers *Ki67*, *Top2a*, and *Cdk1* by 71, 51, and 34-fold respectively (Fig. 3A and Suppl. datasheet 1). Expression of well-known BMP-2 signaling pathways targets such as *Id1*, *Calb1*, and *Hey-1* were upregulated (Fig. 3A and Suppl.1). In order to identify biological pathways affected by BMP-2 we performed Gene ontology (GO) analysis on the differentially expressed genes. BMP-2 upregulated genes were enriched in biological process pathways involved in insulin and peptide transport and secretion pathways and monocarboxylic acid and lipid metabolic processes (Fig. 3C and Suppl. datasheet 2). Of interest, upregulation of the negative regulators of insulin secretion *Ucp2* [48, 49] and *Acvr1c* [50] could explain the decreased insulin secretion capacity observed in BMP-2 exposed islets. BMP-2 downregulated genes were strongly associated with functional pathways of the cell cycle, replication, and cell division (Fig. 3D and Suppl. datasheet 3).

### BMP-2-regulated gene expression correlates with protein expression

**Fig. 4:**
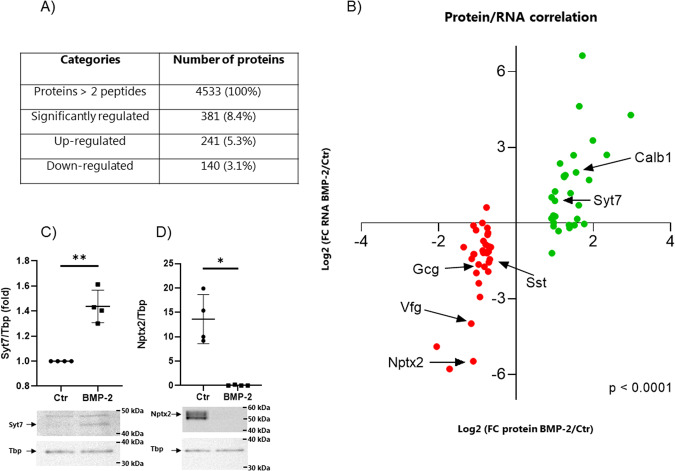
BMP-2 regulated gene expression correlates with protein expression. Mouse islets were exposed to BMP-2 (50 pg/ml) or left non-exposed for 10 days. Mass spectrometry was performed on extracts and **A** show significantly regulated (up or down) proteins (proteins >2 peptides, FDR <0.05, FC >0.3)) in BMP-2 exposed vs. ctr islets, *N* = 5. **B** Correlation plot of Protein vs. RNA regulated by BMP-2. X-axis: Log2 (FC protein BMP-2/ctr), y-axis: Log2 (FC RNA BMP-2/ctr). The top 35 significantly up- and down-regulated proteins are shown. Gray dots represent proteins where the corresponding mRNA was not significantly regulated by BMP-2. Green dots represent proteins where the corresponding mRNA was significantly upregulated by BMP-2 and red dots represent proteins where the corresponding mRNA was significantly downregulated by BMP-2. Spearman correlation test ***p < 0.0001. **C** Nptx2 and **D** Syt7 protein expression was measured in whole cell extracts from mouse islets exposed as described above using Western blotting. Representative blots are shown, and Tbp was used as a loading control. Band intensities were quantified using Image Studio and data were shown as fold increase relative to Ctr (Syt7) or as relative A.U. expression (Nptx2). Data show means ± SD of *N* = 4. *p < 0.05, **p < 0.01, two-sided paired *t*-test vs ctr.

Next, we investigated whether BMP-2-regulated mRNA expression correlated with protein expression. A global proteomic analysis was performed on mouse islets exposed to BMP-2 or left non-exposed for 10 days. BMP-2 exposure led to significant up- and downregulation of 211 and 131 proteins, respectively (protein >2 peptides, adj. *P* value >0.05, FC >0.3) (Fig. 4A, Suppl. Fig. 1, and Suppl. datasheet 4,5). A top 35 most up- or downregulated proteins list was generated with corresponding mRNA expression (Table 1). We found a significant correlation between BMP-2 protein product and mRNA expression (Fig. 4B). In accordance with the mRNA-seq data, we previously showed that the CALB1 protein level was significantly upregulated by BMP-2 [24]. We also verified some of the top 35 most BMP-2 regulated proteins by Western blotting. Syt7 protein was significantly upregulated in islets exposed to BMP-2 for 10 days whereas Nptx2 protein expression was significantly downregulated (Fig. 4C, D and Supplementary original files). No significant changes were observed in INS1, MAFA, and PDX1 protein expression after BMP-2 stimulation. GO analysis showed that only one biological process pathway upregulated upon BMP-2 stimulation, the regulation of system processes (Suppl. datasheet 6), and one downregulated protein pathway related to the endoplasmic reticulum (ER) stress (Suppl. datasheet 7). Of importance, we did also observe a significant GO term related to ER stress at the mRNA level (Suppl. datasheet 3). A STRING analysis on BMP-2 downregulated proteins (FC >0.5) further revealed highly confident networks of protein processing in ER and signal peptide processing. Interestingly, and supporting our GO analysis on the cell cycle, protein networks associated with DNA replication minichromosome maintenance protein complex (MCM) important for cell division and various checkpoint pathways were also identified (Suppl. Fig. 2).

**Table 1:** Top 35 most regulated proteins and corresponding genes expression by BMP-2.

	Gene name	Protein name	Abundance protein Ratio: (BMP2) / (Control)	Abundance protein Ratio Adj. *P*-Value: (BMP2) / (Control)	Abundance mRNA Ratio: (BMP2) / (Control)	Abundance mRNA Ratio Adj. *P*-Value: (BMP2) / (Control)
**Top 35 most upregulated proteins and corresponding genes by BMP2:**						
1	Abcg2	Broad substrate specificity ATP-binding cassette transporter ABCG2	7,83	0,000001	19,46	0,000618
2	H1-4	Histone H1.4	5,81	0,005678	N.A	N.A
3	Ca15	Carbonic anhydrase 15	5,10	0,000066	6,49	0,000618
4	Mxra7	Matrix-remodeling-associated protein 7	3,97	0,000062	9,64	0,000618
5	S100a6	Protein S100-A6	3,71	0,019002	3,26	0,013735
6	Rpl14	60 S ribosomal protein L14	3,40	0,074282	0,97	0,894231
7	Apoa5	Apolipoprotein A-V	3,29	0,000142	99,64	0,000618
8	Krt20	Keratin, type I cytoskeletal 20	3,11	0,020971	24,75	0,377516
9	Vstm2l	V-set and transmembrane domain-containing protein 2-like protein	3,08	0,007263	1,63	0,000618
10	Dap	Death-associated protein 1	3,00	0,004513	1,12	0,401089
11	Calb1	Calbindin	2,93	0,000001	4,01	0,000618
12	Lsm6	U6 snRNA-associated Sm-like protein LSm6	2,86	0,029078	0,94	0,748914
13	Iqgap2	Ras GTPase-activating-like protein IQGAP2	2,81	0,000003	6,42	0,000618
14	Spock2	Testican-2	2,66	0,000031	2,27	0,000618
15	Rps20	40 S ribosomal protein S20	2,61	0,032729	0,87	0,331214
16	H2ac20	Histone H2A type 2-C	2,60	0,016075	N.A	N.A
17	Rpl34	60 S ribosomal protein L34	2,59	0,052033	0,95	0,957406
18	Tppp3	Tubulin polymerization-promoting protein family member 3	2,42	0,048060	3,72	0,000618
19	Gsta1	Glutathione S-transferase A1	2,41	0,081305	N.A	N.A
20	Anxa10	Annexin A10	2,37	0,026457	3,55	0,119353
21	Ptgr1	Prostaglandin reductase 1	2,37	0,009449	3,64	0,000618
22	Isoc1	Isochorismatase domain-containing protein 1	2,25	0,056079	N.A	N.A
23	Igfbp5	Insulin-like growth factor-binding protein 5	2,20	0,005785	5,13	0,000618
24	Gpr180	Integral membrane protein GPR180	2,14	0,028357	0,80	0,043977
25	Osbpl10	Oxysterol-binding protein-related protein 10	2,08	0,000083	N.A	N.A
26	Acyp1	Acylphosphatase-1	2,02	0,029866	1,20	0,272694
27	Sult1d1	Sulfotransferase 1 family member D1	2,02	0,001639	2,38	0,000618
28	Syt7	Synaptotagmin-7	2,01	0,000264	1,84	0,000618
29	Dpy30	Protein dpy-30 homolog	1,97	0,064369	1,01	0,966752
30	Uevld	Ubiquitin-conjugating enzyme E2 variant 3	1,96	0,060962	0,92	0,784857
31	Zdhhc2	Palmitoyltransferase ZDHHC2	1,96	0,001802	1,23	0,048838
32	Krt19	Keratin, type I cytoskeletal 19	1,91	0,073123	0,44	0,012744
33	Ces2c	Acylcarnitine hydrolase	1,90	0,050217	N.A	N.A
34	S100a1	Protein S100-A1	1,89	0,002433	2,03	0,000618
35	Sumo1	Small ubiquitin-related modifier 1	1,89	0,025861	1,13	0,424157
**Top 35 most downregulated proteins and corresponding genes by BMP2:**						
1	Stmn1	Stathmin	0,24	0,000052	0,03	0,198626
2	C1qa	Complement C1q subcomponent subunit A	0,30	0,000045	0,02	0,000618
3	Ppp4r4	Serine/threonine-protein phosphatase 4 reg. subunit 4	0,39	0,007247	0,51	0,000618
4	Vgf	Neurosecretory protein VGF	0,45	0,000007	0,06	0,000618
5	Psat1	Phosphoserine aminotransferase	0,45	0,000213	0,37	0,000618
6	Nptx2	Neuronal pentraxin-2	0,47	0,000305	0,02	0,000618
7	Eef1b	Elongation factor 1-beta	0,47	0,076116	0,93	0,647443
8	Mcm2	DNA replication licensing factor MCM2	0,47	0,000095	0,42	0,000618
9	Mcm6	DNA replication licensing factor MCM6	0,47	0,000153	0,42	0,000618
10	Eri1	3'-5' exoribonuclease 1	0,49	0,037395	0,82	0,174334
11	Nefm	Neurofilament medium polypeptide	0,49	0,000197	0,25	0,000618
12	Gcg	Pro-glucagon	0,51	0,000596	0,32	0,000620
13	Prom1	Prominin-1	0,51	0,000317	0,19	0,000618
14	Mcn3	DNA replication licensing factor MCM3	0,53	0,000420	0,13	0,000618
15	Rif1	Telomere-associated protein RIF1	0,55	0,010896	1,00	0,980331
16	Dgkg	Diacylglycerol kinase gamma	0,55	0,000732	0,47	0,000618
17	Dmd	Dystrophin	0,56	0,024898	0,61	0,007054
18	Dbn1	Drebrin	0,56	0,000064	0,44	0,000618
19	Creld2	Protein disulfide isomerase Creld2	0,56	0,001105	0,45	0,000618
20	Gpx3	Glutathione peroxidase 3	0,57	0,007145	0,30	0,000618
21	Eppk1	Epiplakin	0,57	0,000756	0,45	0,034942
22	Cgref1	Cell growth regulator with EF hand domain protein 1	0,58	0,000560	0,55	0,836251
23	Rps25	40 S ribosomal protein S25	0,59	0,002133	1,53	0,269735
24	Dync1i1	Cytoplasmic dynein 1 intermediate chain 1	0,60	0,000162	0,44	0,000618
25	Ap1s1	AP-1 complex subunit sigma-1A	0,60	0,000025	0,44	0,000618
26	Nudt7	Peroxisomal coenzyme A diphosphatase NUDT7	0,60	0,000271	0,87	0,411039
27	Tmem165	Transmembrane protein 165	0,60	0,004953	0,72	0,001174
28	Ephx1	Epoxide hydrolase 1	0,61	0,000104	0,76	0,017847
29	Rassf2	Ras association domain-containing protein 2	0,61	0,001264	0,26	0,000618
30	Erich5	Glutamate-rich protein 5	0,61	0,000294	0,53	0,062129
31	Spock1	Testican-1	0,61	0,016193	0,46	0,000618
32	Enpp2	Ectonucleotide phosphodiesterase family member 2	0,62	0,000153	0,34	0,000618
33	Sst	Somatostatin	0,63	0,075366	0,37	0,000618
34	Oat	Ornithine aminotransferase, mitochondrial	0,63	0,000717	0,52	0,000618
35	Rcn1	Reticulocalbin-1	0,64	0,000089	0,52	0,000618

Table showing the top 35 most regulated proteins with corresponding mRNA expression and related adjusted p-value. N.A= no corresponding mRNA detected.

### BMP-2 regulates histone modifications

**Fig. 5:**
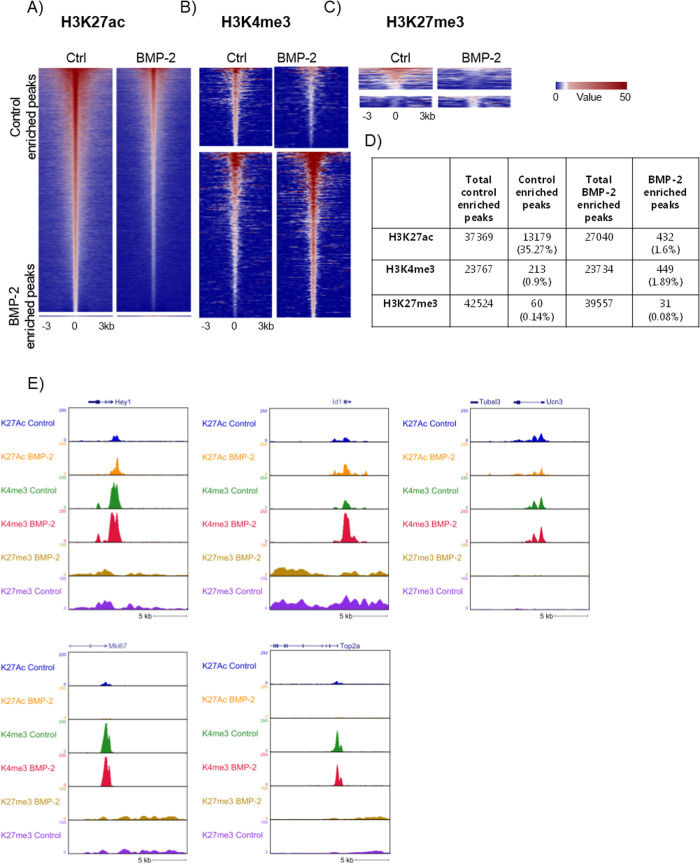
BMP-2 regulation of histone modifications. Analysis of chromatin histone modification in mouse islets exposed to BMP-2 (50 pg/ml) or left non-exposed for 10 days. Heatmap of **A** H3K27 acetylation, **B** H3K4 tri-methylation, and **C** H3K27 tri-methylation from Ctr (left) and BMP-2 exposed islets (right) from −3 kb to +3 kb surrounding the center of the peak. Each horizontal line represents a single histone 3 binding site (peak) and the color scale indicates the H3K27ac, H3K4me3, and H3K27me3 ChIP-seq tags per bp, respectively. H3K27ac scale is 5E-5 cm per peak while for H3K4me3 and H3K27me3 the scale is 0.01 cm per peak. Upper panel: control enriched peaks (downregulated by BMP-2). Lower panel: BMP-2 enriched peaks (upregulated by BMP-2). **D** Table showing the summary of the number of peaks regulated by BMP-2. **E** Genome browser snapshot of ChIP-seq for selected genes; *Hey-1, Id1, Ucn3, Ki67*, and *Top2a* for the 3 histone modifications (H3K27ac, H3K4me3, and H3K27me3) from ctr and BMP-2 exposed islets.

We next investigated whether changes in gene expression induced by BMP-2 were associated with histone modifications. We analyzed three specific modifications, H3K27ac, H3K4me3, and H3K27me3 by chromatin immunoprecipitation followed by sequencing (ChIP-seq) using chromatin preparations from isolated islets cultured in the absence or presence of BMP-2 for 10 days. Of the three modifications analyzed, we found H3K27ac to be the most affected by BMP-2 where a total of 13,611 regulated peaks (FDR Poisson *P* value <0.0001 and FC >2) were identified with 13,179 peaks found to be enriched in the control (down by BMP-2) and 432 peaks were found to be enriched in the BMP-2 exposed conditions (Fig. 5A, D). For H3K4me3, a total of 662 regulated peaks (FC >2) were identified with 213 peaks enriched in the control and 449 peaks increased by BMP-2 exposure (Fig. 5B, D). For H3K27me3, a total of 91 peaks were regulated with 60 and 31 peaks (FC >2) found to be enriched in the control or BMP-2 exposed cells, respectively (Fig. 5C, D) (List of differential peaks, Suppl. datasheet 8–13).

We hypothesized that BMP-2 exposure leads to a reduction of H3K27ac near the transcription start site (TSS) of mature beta cell genes, leading to decreased transcription. We found that the beta cell marker *Ucn3* as well as the proliferation markers *MKi67* and *Top2a* had lower levels of H3K27 acetylation in the proximity to their respective TSS while the BMP-2 enhanced genes *Id1* and *Hey-1* showed increased H3K27ac marks at the TSS (Fig. 5E). This is in agreement with the mRNA expression data. Furthermore, an increase in H3K4 tri-methylation was found in the vicinity of the *Id1* gene (Fig. 5E).

**Fig. 6:**
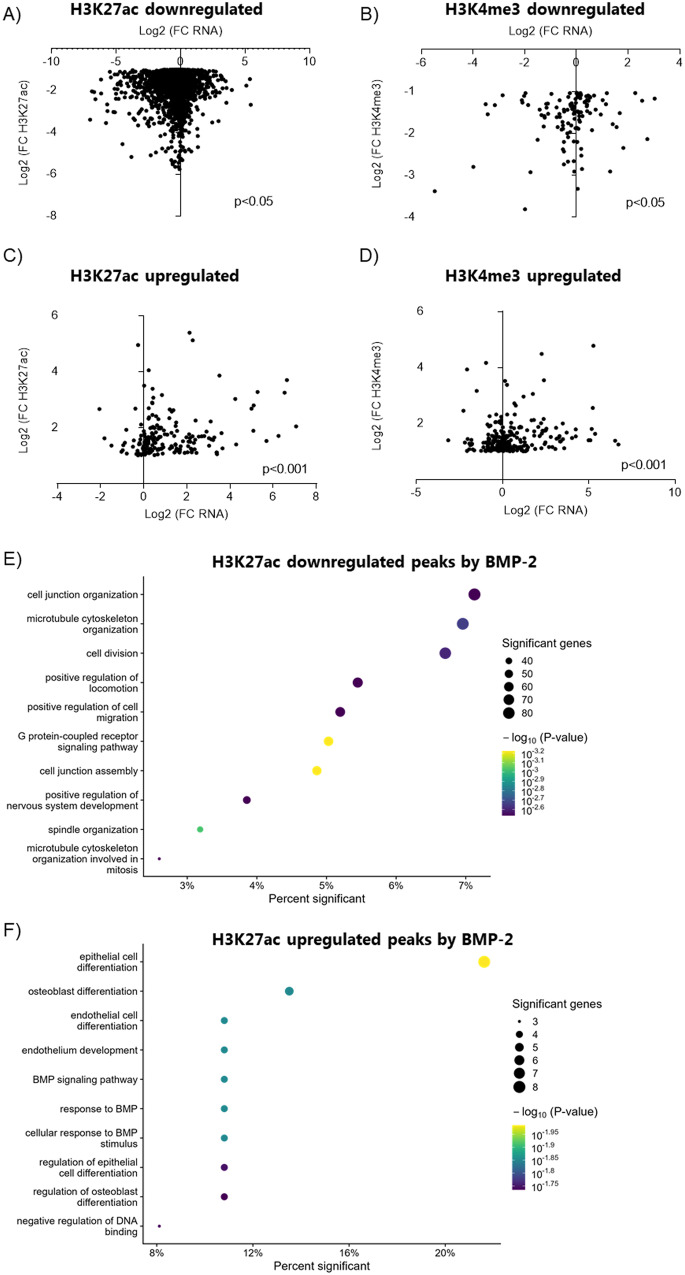
BMP-2-induced changes in RNA expression are associated with changes in histone modifications. Graph showing the correlation between RNA fold-change and **A** H3K27ac Ctr enriched peaks. **B** H3K4me3 Ctr enriched peaks. **C** H3K27ac BMP-2 enriched peaks. **D** H3K4me3 BMP-2 enriched peaks. X-axis: Log2 (FC RNA) Y-axis: Log2 (FC histone modification). *p < 0.05, ***p < 0.001 Spearman correlation test. **E** Biological process GO analysis on H3K27ac downregulated peaks associated with TSS of genes. **F** Biological process GO on H3K27ac BMP-2 upregulated peaks associated with TSS of genes. X-axis: Percent of genes in the GO with a differential peak associated with TSS. Circle: Number of genes in the GO with a BMP-2 regulated differential peak associated with their TSS.

As H3K27ac and H3K4me3 were the histone modifications found to be most strongly affected by BMP-2, we next analyzed the link between differentially regulated histone signals (H3K27ac and H3K4me3 associated with TSS of annotated genes) and mRNA expression (Fig. 6A–D). Downregulation of mRNA expression was significantly correlated with a decrease in H3K27ac in control enriched peaks (Fig. 6A) while an upregulation of mRNA expression by BMP-2 was significantly correlated with an increase in H3K27ac (Fig. 6C). A similar correlation was found for H3K4me3; a decrease in mRNA expression was significantly correlated with a decrease in H3K4me3 (Fig. 6B) while an increase in gene expression by BMP-2 was significantly correlated with an increase in H3K4me3 (Fig. 6D). Accordingly, genes proximal to differential H3K27ac or H3K4me3 sites following BMP-2 treatment exhibited concordant changes in mRNA levels, with the exception of sites losing H3K4me3 (Suppl. Fig. 3A–D). We further performed GO analysis of genes with significantly regulated peaks in their promoters. Several significant GO Biological processes were identified in H3K27ac downregulated peaks, many of which related to proliferation and cell cycle regulation and organization (Fig. 6E and Suppl. datasheet 15). H3K27ac upregulated peaks by BMP-2 were associated to GO terms related to BMP response and signaling and differentiation of various cell types (Fig. 6F and Suppl. datasheet 14). No Biological Processes GO terms were significant for H3K4me3 peak-associated genes (Suppl. datasheet 16, 17).

### BMP-2 stimulation leads to a decrease in NeuroD1 binding to chromatin

**Fig. 7:**
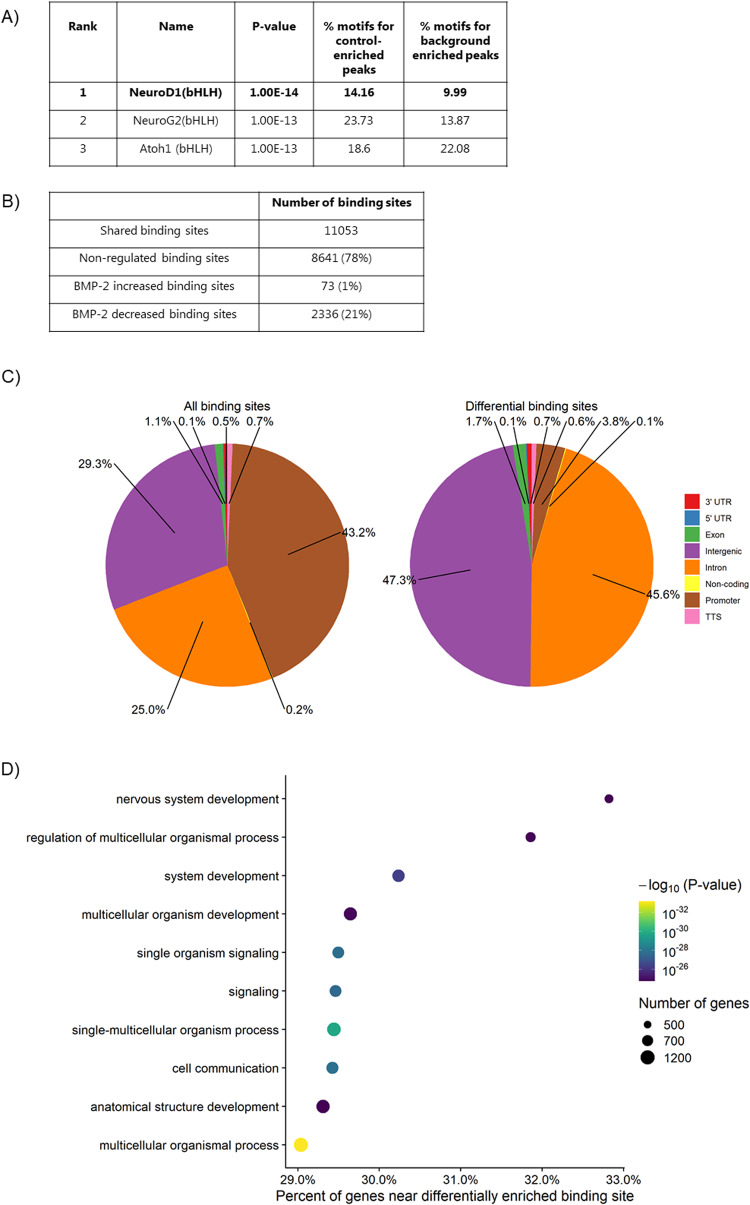
BMP-2 induced regulation of NeuroD1 chromatin binding. **A** Table depicting the top three enriched motifs in H3K27ac Control enriched (downregulated by BMP-2) peaks. **B** ChIP analysis showing the number of NeuroD1 binding sites (peaks) upregulated and downregulated by BMP-2. **C** Distribution of NeuroD1 occupied binding sites in the genome and differential NeuroD1 binding sites in response to BMP-2. **D** Biological processes GO analysis on BMP-2 downregulated NeuroD1 binding sites. X-axis: Percent of all genes in GO, that are near a differentially enriched binding site. Circle: Number of genes in the GO close to a differentially enriched binding site.

Motif analysis (HOMER) of H3K27ac peaks revealed NeuroD1 and other bHLH family members binding sites within H3K27ac peaks downregulated by BMP-2 (Fig. 7A). No statistically significant motifs were found in BMP-2 regulated H3K4me3 peaks. Since Id proteins are known to dimerize with other bHLH factors such as NeuroD1, preventing their binding to DNA, we analyzed if the decrease in H3K27ac peaks was associated with a decrease in NeuroD1 binding. NeuroD1 ChIP analysis showed that BMP-2 reduced NeuroD1 binding to chromatin. A total of 11,053 NeuroD1 binding sites were identified, of those, 2412 binding sites showed a significant change (FDR Poisson *P* value <0.0001, FC >2) upon BMP-2 exposure. Of these 2336 peaks showed decreased binding and only 76 peaks showed increased binding following BMP-2 exposure (Fig. 7B). As expected, NeuroD1 was found to bind in the promotors of key beta cell genes such as *Ins1/2* and *Pdx1*. However, no significant differences in the binding to these specific sites were observed between control and BMP-2 exposed islets. Instead, we found a significant reduction of NeuroD1 binding in the promotors of the beta cell markers *MafA*, *Ucn3*, and *FoxO1* (a list of differential peaks can be found in Suppl. datasheet 18, 19). Comparison of the distribution of NeuroD1 binding sites in the genome demonstrated that differently occupied sites were depleted of promoters and enriched in intergenic and intronic regions in response to BMP-2 (Fig. 7C). Biological Processes GO analysis revealed that BMP-2 downregulated NeuroD1 binding was enriched in gene sets of organismal processes, development, and signaling (Fig. 7D and Suppl. datasheet 20).

## Discussion

In this study we have explored the changes in function, gene and protein expression, and histone modifications in mouse islets exposed to BMP-2, to better understand the mechanism behind BMP-2-induced beta cell dysfunction.

Beta pancreatic cells exposed to BMP-2 showed decreased insulin release in response to glucose, which was associated with a marked reduction in mRNA expression of genes controlling beta cell function such as *Ins1* and *Ucn3* and upregulation of Calbindin1 (*Calb1*). Changes in *Ins1* expression did not translate into decreased insulin protein expression, when analyzed by proteomics or ELISA from islet extracts. However, in contrast to the insulin expression data, our top 35 protein expression analysis on BMP-2 exposed mouse islets showed a significant correlation between the regulation of mRNA and protein expression. Several genes and corresponding proteins in our top 35 table are involved in vesicle trafficking and exocytosis, including Calb1, Syt7, Vgf and Nptx2 indicating an overall effect of BMP-2 on vesicle trafficking and exocytosis. We have previously shown that BMP-4 reduced exocytosis through a reduction in Ca^2+^ current through voltage-dependent Ca^2+^ channels, independent of insulin content. This effect was suggested to be mediated via a BMP-4-induced increase in Calb1 mRNA and protein expression, indicating an effect of BMP-4 directly on insulin secretion [24].

The BMP-2-induced beta cell dysfunction was also associated with an increased expression of the bHLH family members *Id1-3*, *Hey-1*, and *Hes-1* which are negative regulators of bHLH transcription factor activity [25, 27]. The Notch signaling pathway is known to induce Hes-1 activation, but is largely inactive in adult pancreas tissue. However, under certain conditions associated with dedifferentiation (e.g., in vitro culture and cytokine exposure) Notch signaling can be re-activated [32, 51]. The upregulation of Hes-1 in cultured human islets was associated with an increase in beta cell proliferation and dedifferentiation [32]. Moreover, inhibition of insulin expression by Hes-1 has been suggested to be due to Hes-1 direct binding to NeuroD1 or Pdx1 thus decreasing *Ins* gene transcription [33, 51]. However, an increase in *Hes-1* mRNA in this study was not associated with an increase in proliferation.

Beta cell proliferation was significantly reduced by BMP-2 exposure. The top ten gene ontology of biological processes downregulated by BMP-2 showed an enrichment of genes associated with cell proliferation, cell cycle division, and chromosome segregation, indicating that BMP-2 might exert its effects on proliferation by inhibiting the progression of the G2/M phase. Of interest, we found decreased expression of *Cdk1*, *Ki67*, *Cenpf*, and *Top2a*, which are markers of replicating beta cells [52]. Further, STRING analysis of BMP-2 downregulated proteins revealed decreased expression of several proteins in the MCM complex critical for DNA replication [53] and glucose-induced proliferation [54]. G1/S CDK complexes play a crucial role in beta cell proliferation in mouse and human, and loss of either CDK4 or Cyclin D2 leads to a dramatic loss of beta cell mass and proliferation, resulting in diabetes [55, 56]. CDK6 alone or in combination with a D-Cyclin can drive human beta cell replication in vitro and in vivo [57, 58]. Furthermore, nutrients and growth factors have been shown to induce beta cell replication by inducing the progression of G1/S [59, 60]. In this study, we have not found differences in the mRNA or protein expression of *Cdk4*, *Cdk6*, or *Ccnd2*. However, reduced mRNA expression of *Ccnd1* was observed. CCND1 forms a complex with CDK4, E2F1, and E2F2, which are both activators of the G1/S transition and are significantly reduced by BMP-2. In addition, overexpression of Hes-1 has been shown to arrest the human breast T47D cells in the G1 phase of the cell cycle by repressing the expression of *E2F1* [61]. Altogether, this data suggests that BMP-2 inhibits proliferation by inhibiting the progression of the G1/S and G2/M phase.

Epigenetic regulation is essential for proper function including cell proliferation. We analyzed the BMP-2-induced changes in three histone marks known to affect chromatin structure and gene transcription in islet cells. The observed reduction in the H3K27ac mark following BMP-2 exposure and the association with cell cycle genes (e.g., *Top2a*, *Mki67*, and *E2f2*) suggest that this histone modification is significant and plays a role in the inhibition of beta cell proliferation induced by BMP-2. In agreement with RNA-seq data, *Ucn3* also show reduced H3K27ac in the proximity to their TSS, while *Pdx1* showed an increase. Furthermore, *Pdx1* and *Glp1r* genes exhibited an increase in the H3K4me3 mark. Both H3K27ac and H3K4me3 marks have previously been found to be associated with several beta cell-specific genes in human and mouse islets, indicating that they might play an important role in the regulation of transcription of these genes [43, 62–64].

Motif analysis of BMP-2 downregulated H3K27ac peaks revealed enrichment in NeuroD1 binding sites and other bHLH family members. NeuroD1 ChIP-seq confirmed that BMP-2 stimulated cells had decreased NeuroD1 binding to chromatin and GO analysis of BMP-2 downregulated binding sites revealed significant enrichment of genes involved in development. Both the Id proteins and Hes-1/Hey-1 inhibit the transcriptional activity of bHLH factors. The Id proteins, which lack a DNA binding domain, dimerize with bHLH transcription factors such as NeuroD1 to prevent their binding to DNA and thereby reduce transcription [25]. On the other hand, Hes-1 and Hey-1 bind E- and N- box sequences as homo- or heterodimers and act as transcriptional repressors. Hes-1/Hey-1 can also form dimers with bHLH transcription factors and change the transactivating activity to repression while maintaining DNA binding activity [65]. The fact that BMP-2 stimulation caused a general decrease in NeuroD1 chromatin binding without affecting the NeuroD1 mRNA level or protein expression suggests a model in which Id proteins prevent NeuroD1 binding by forming Id/NeuroD1 dimers. However, a role for Hes-1/Hey-1 to heterodimerize with NeuroD1 and subsequent recruitment of transcriptional repressors could also be at play. Further analysis of the Id and Hes-1/Hey-1 binding partners in BMP-2 stimulated islets is required to characterize the mechanism in more detail. Hey-1 proteins have been shown to directly interact with the co-repressors N-CoR and mSin3A, which in turn are able to recruit histone deacetylase-1 (HDAC1) and induce gene silencing [46]. Hes-1 has also been described to recruit another histone deacetylase, SIRT1 [47]. We speculate that the recruitment of co-repressor by Hes-1/Hey-1 might induce the observed reduction of H3K27ac upon BMP-2 exposure.

In conclusion, in this study, we show that prolonged exposure to BMP-2 is associated with beta cell dysfunction and inhibition of proliferation. We provide evidence that BMP-2 exposure leads to a reduction in H3K27ac, which is associated with reduced expression of positive regulators of the cell cycle. BMP-2 exposure was also associated with a decrease in NeuroD1 binding, indicating that Id proteins might be the effectors of beta cell dysfunction induced by BMP-2.

## Methods and materials

### Mouse islet isolation and culture

Pancreata from 12-week-old male C57BL/6NJR mice (Janvier, Saint Berthewin Cedex, France) or 12-week-old male C57BL/6NTAC mice (Taconic, Lille Skensved, Denmark) were inflated with Liberase (Roche, Hvidovre, Denmark), excised and incubated at 37 °C to allow digestion. Digestion was halted by adding Hanks’ balanced salt solution (HBSS; Lonza, Basel, Switzerland) supplemented with bovine serum albumin (BSA; Sigma, Soeborg, Denmark). Islets were handpicked and cultured for 1 day in 10 ml RPMI 1640 medium (Lonza) with 11 mmol/l d-Glucose supplemented with 10% fetal bovine serum (FBS; Biosera, Herlev, Denmark) and 1% penicillin/streptomycin (100 U/ml penicillin, 100 μg/ml streptomycin) (P/S; Gibco, Life Technologies, Roskilde, Denmark). Thereafter islets were cultured for 10 days at 37 C° in a humidified atmosphere with 5% CO_2_ in 10 ml RPMI 1640, 2% human serum (HS; Lonza), and 1% P/S in the absence or presence of 50 ng/ml recombinant human BMP-2 (R&D Systems, Life Technologies). The medium was changed every fifth day. For GSIS, apoptosis and proliferation analysis, islets from individual mice were used. For RNA isolation, Proteomic and ChIP-seq analysis, pools of islets from several mice were used. All animal experiments were approved by the local ethics committee, and animals were housed according to the Principals of Laboratory Care.

### Glucose-stimulated insulin release and insulin content

GSIS was performed with 25 islets per condition in biological duplicates as previously described (*N* = 5) [66]. In brief, islets were pre-cultured for 1.5 h in Krebs–Ringer HEPES buffer (KRHB; 115 mmol/l, NaCl, 4.7 mmol/l KCl, 2.6 mmol/l CaCl2, 1.2 mmol/l KH2PO4, 1.2 mmol/l MgSO4, 10 mmol/l HEPES, 0.2% BSA, 2 mmol/l glutamine, 5 mmol/l NaHCO3, and 1% penicillin and streptomycin, pH 7.4) with 2 mM d-glucose followed by 30 min of incubation in KRHB with 2 mM d-glucose and then 30 min of incubation in KRBH with 20 mM d-glucose. The buffer was then collected from each experimental group and insulin content was determined using an in-house ELISA. Results were corrected for DNA content using Quant-IT PicoGreen dsDNA Reagent and Kit (Life Technologies).

### Proliferation

Beta cell proliferation was analyzed in intact mouse islets cultured on extracellular matrix-coated slides (Biological Industries, Israel). About 10 uM EdU (ethynyl-2’-deoxyuridine) (Life Technologies) was given 24 h prior to fixation in 1% PFA. Proliferating beta cells were detected with Click-iT EdU Proliferation Assay (Life Technologies), followed by staining of pancreatic duodenal homeobox 1 (Goat-anti Pdx1; 1:5000, ab2027, Beta cell biology consortium) and Hoechst (#3342, Life Technologies). Islet cells are positive for both Pdx1 and EdU were counted as proliferating beta cells. Whole islets were examined by capturing z-stack images using Zeiss Axio Observer Z1 with a spinning disk (Zeiss, Birkerød, Denmark) and counted using the ZEN software (Zeiss). A minimum of 700 cells were examined per experiment in each condition (*N* = 4). For representation, a median filter with binding 2 × 2 pixels was applied (Image J).

### Cell death detection assay

Apoptotic cell death was measured in 25 islets per condition in biological duplicates (*N* = 3) by the detection of DNA histone complexes released from the nucleus to the cytosol using Cell Death Detection ELIS APLUS (Roche) as described by the manufacturer. As a positive control for apoptosis mouse islets were exposed for 48 h to 300 pg/ml recombinant mouse IL-1β (cat. 554577, BD Biosciences, Lyngby, Denmark) combined with 10 ng/ml IFN-γ (cat. 585-IF, R&D System, Life Technologies). To adjust for differences in cell number among different conditions, islet lysates were sonicated and DNA was quantified by Quant-IT PicoGreen dsDNA Reagent and Kit (Life technologies).

### Caspase 3/7 activity

For measurement of caspase 3/7 activity, the Caspase-Glo 3/7 Assay System (Promega Biotech AB, Nacka, Sweden) was used according to the manufacturer’s protocol. About 12 size-matched islets in biological duplicate per condition were cultured as described (*N* = 4).

### Gene expression analysis

A total of 200–400 intact mouse islets were cultured as described and lysed in Trizol (*N* = 12). Total mRNA purification was performed using a Direct-zol RNA-mini prep kit according to the manufacturer’s protocol (Zymo Research, Nordic Biosite, Copenhagen, Denmark). cDNA was synthesized by qScript cDNA Supermix kit (Quanta Biosciences, Beverly, MA, USA). Quantitative expression levels of mRNA of interest were evaluated using Taqman Gene Expression probes and performed on an ABI PRISM 7900HT Sequence Detection system (Applied Biosystems, Nærum, Denmark). Each sample was measured in triplicates and expression was normalized to the internal control, Ppia. The TaqMan Gene Expression probes against Ins1 (Mm01950294_s1), Ins2 (Mm00731595_gh), Mafa (Rn00845206_s1), Ucn3 (Mm00453208), Pdx1 (Mm00435565), Glp1R (00445292_m1), Hes-1 (Rn00577566_m1), Hey-1 (Rn00448865_m1), Ki67 (Mm01278617_m1), Cdk1 (Mm00772472-m1), and Ppia (Rn00690933_m1) were used for quantifying mRNA expression of the different genes.

### RNA-seq

RNA was isolated from 500 islets per condition (*N* = 3) using the RNeasy® Micro Kit (Qiagen) according to the manufacturer’s instructions. For RNA sequencing (RNA-seq), stranded, single-end sequencing libraries were constructed from 35 ng of isolated RNA using the TruSeq® Stranded mRNA Library Prep Kit (Cat# 20020595, Illumina®, San Diego, CA, USA). Library sequencing was performed on a HiSeq 4000 System (Illumina®). Both library construction and sequencing were performed by the IGM core research facility at the University of California San Diego, USA.

Single-end 50 bp RNA-seq reads were mapped to the UCSC mouse genome (mm9) by STAR v. 2.4.0 (--outSAMstrandField intronMotif--outFilterMultimapNmax 1 --rethread 5), allowing for up to ten mismatches (default by STAR) [67]. Only the reads aligned uniquely to one genomic location were retained for subsequent analysis. Expression levels of all genes were estimated by Cufflinks v2.2.1 (-p 6 -G $gtf_file --max-bundle-frags 1000000000) [68] using only the reads with exact matches. Differential gene expression was performed using Cuffdiff (Cufflinks v2.2.1), with FDR <0.05 considered significant.

RNA-seq data from control islets are identical to the data set published previously as these samples served as controls for both BMP-2 stimulation (this study) and IL-1ß stimulation [69]. This was done in an effort to comply with the 3R principles for animal research.

To identify the functional categories of differentially expressed genes, GO analysis of Biological Processes was performed using the enrichGO function from the R package clusterProfiler v. 4.4.4 [70] using the org.Mm.eg.db v 3.15.0 database [71] with all expressed genes as the universe (background) and genes with an FDR less than 0.05 as significant genes (foreground), only terms containing between 10 and 500 expressed genes were analyzed. GO analysis was performed separately for genes that were up- or downregulated. Dot plots were generated using the dotplot function, which is part of clusterProfiler. The color was mapped to –log10 (*P* value) instead of raw *P* value. Ontologies with an FDR less than 0.05 was considered significant and the top ten most significant terms were visualized in the dot plots. Suppl. Tables of ontologies include non-significant ontologies with a more lenient FDR cutoff of 0.5.

### Proteomics

About 150 intact mouse islets per condition (*N* = 5) were collected and washed x3 in phosphate-buffered saline (PBS). Islets for the proteomics workflow were lysed, reduced, and pre-digested in 10 µl lysis buffer (6 M Urea, 2 M Thiourea, and 10 mM DTT) (Sigma) and 1 µg Lys-C (Wako) supplemented with PhosSTOP phosphatase inhibitor (Roche) for 2 h at RT. Then lysates were diluted ten times using 20 mM Triethylammonium bicarbonate buffer (TEAB; pH 8) and tip-sonicated for 2 × 10 s on the ice at 60% amplitude. Then, samples were alkylated by 20 mM Iodacetamide for 20 min in the dark before digestion with 2% (w/w) trypsin (Sigma) overnight (ON) at 30 °C.

A total of 100 µg tryptic peptides from each sample were labeled with TMT10 plex according to the manufacture’s protocol (Controls: TMT126-TMT128C; BMP-2: TMT129N-TMT131). After labeling, the samples were mixed in a 1:1 ratio and the sample was dried by vacuum centrifugation. The lyophilized sample was re-solubilized in 0.1% TFA and an aliquot corresponding to 100 µg peptide was desalted using a micro Oligo R3 (Applied Biosystems) Reversed-phase column as described previously [72]. The desalted peptide sample was dried by vacuum centrifugation.

### Hydrophilic interaction liquid chromatography (HILIC)

The peptide samples were subjected to fractionation using HILIC. Briefly, these samples were resuspended in 90% ACN, 0.1% TFA (Solvent B), and loaded onto a 450 µM OD × 320 µM ID × 17 cm micro-capillary column packed with TSK Amide-80 resin material (Tosoh Bioscience) using an Agilent 1200 Series HPLC (Agilent). Peptides were separated using a gradient from 100–60% Solvent B (Solvent A: 0.1% TFA) running for 30 min at a flow rate of 6 µL/min. Fractions were collected every 1 min and combined into 13 final fractions based on the UV chromatogram and subsequently dried by vacuum centrifugation.

### Reversed-phase nanoLC-ESI-MS/MS

The samples were resuspended in 0.1% TFA and loaded onto an EASY-nLC system (Thermo Scientific). The samples were loaded onto a two-column system containing a 3 cm pre-column and a 17 cm analytic column both consisting of fused silica capillary (75-μm-inner diameter) packed with ReproSil – Pur C18 AQ 3 μm reversed-phase material (Dr. Maisch). The peptides were loaded onto the pre-column in buffer A (0.1% formic acid (FA)). The peptides were eluted with an organic solvent gradient from 1% buffer B (95% ACN, 0.1% FA) to 7% buffer B at a constant flow rate of 250 nl/min for 3 min and then up to 28% buffer B for 55 min, up to 40% buffer B in 3 min and finally to 95% buffer B in 3 min. The nanoLC was online connected to an Orbitrap Fusion Lumos (Thermo Fisher Scientific) operated at positive ion mode with a data-dependent acquisition. The Lumos acquired the full MS scan with an automatic gain control (AGC) target value of 1 × 106 ions and a maximum fill time of 50 ms. Each MS scan was acquired at high resolution (120,000 full-width half maximum (FWHM)) at m/z 200 in the Orbitrap with a mass range of 400–1600 Da. The Lumos was set to one full MS per 3 s to allow for a maximum amount of precursor ions to be selected for fragmentation in these 3 s. The most abundant peptide ions were selected from the MS for higher energy collision-induced dissociation (HCD) fragmentation (normalized collision energy: 40 V). Fragmentation was performed at high resolution (50,000 FWHM) for a target of 1 × 105 and a maximum injection time of 100 ms using an isolation window of 1.2 m/z and a dynamic exclusion of 20 s. All raw data were viewed in Xcalibur v 3.0 (Thermo Fisher Scientific).

### MS data processing and statistical analysis

All LC-MSMS raw data files were searched using Proteome Discoverer version 2.1. The raw data were searched in PD using a workflow where the raw data were first subjected to database searching using an in-house Mascot server (Version 2.2.04, Matrix Science Ltd., London, UK) and then the spectra that did not yield a high confident identification was further searched using Sequest HT. The searches had the following criteria: database, SwissProt mouse protein database (updated 2017-10) for the Mascot search and UniProt mouse (version 17.07.2017; 75193 sequences) for the Sequest HT search; enzyme, trypsin; maximum missed cleavages, 2; fixed modifications, TMT6plex (N-terminal), TMT6plex (K) and Carbamidomethyl (C). The TMT10 plex reporter ion signals were quantified using S/N and they were normalized to the total S/N in the PD program. After the database search the protein with normalized abundances were extracted and the proteins with two or more identified peptides were used for further analysis. The statistically significant regulated proteins were selected using the program Perseus [73] using multiple sample testing (ANOVA), Benjamini–Hochberg adj. *P* value (>0.05%), FC (>0.3). Perseus was also used for the generation of PCA plots, heat maps, and cluster analysis. GO was performed as with RNA-seq data, using all proteins with 2 or more identified peptides as foreground and proteins with an FDR less than 0.1 as significant. The analysis was performed on up- and downregulated proteins separately.

### Western blot

A total of 200–400 intact mouse islets were cultured as described, washed in PBS, and lysed in RIPA buffer (150 mM NaCl, 1% IGEPAL CA-630, and 0.5% (w/v) sodium-deoxycholate, 0.1% SDS, 50 mM Tris-HCl pH 8, and 2 mM EDTA) supplemented with cOmplete Mini protease inhibitor cocktail (Roche). The samples were diluted to contain 15–25 ug protein and then mixed with NuPAGE LDS (Life Technologies) sample buffer containing 0.1 M DTT. The samples were heated at 80 °C for 10 min and subsequently loaded on 10% Bis-Tris NuPAGE gels (Life Technologies). After separation, the proteins were transferred to nitrocellulose membranes (Life Technologies). The membranes were blocked in 5% (w/v) skim milk for 1 h and incubated overnight at 4 °C with Nptx2 antibody (1:1000, ab277523, Abcam, Cambridge, Great Britain), Syt7 antibody (1:500, MA5-27654, Invitrogen, Waltham, MA, USA) or Tbp antibody (1:1000, #8515, Cell Signaling, Danvers, MA, USA). Horseradish peroxidase-linked anti-rabbit IgG (1:3000, NA934V, Amersham Biosciences, Amersham, Great Britain) or anti-mouse IgG (1:3000, P0260, Agilent Technologies, Glostrup, Denmark) were used as secondary antibody and incubated with the membrane for 1 h at room temperature. Peroxidase activity was detected by chemiluminescence using the ECL Prime Western Blotting Detection Reagents (RPN2232, Amersham) and imaged on Molecular Imager Chemi Doc XRS+ (Bio-rad, Hercules, CA, USA). Densitometric imaging was performed using (Image lab. 6.1, Bio-rad).

### Histone modification ChIP-seq

About 1200–1500 mouse islets per condition (*N* = 3) were cross-linked for 10 min with 1.11% formaldehyde, quenched with 0.125 M glycine, lysed in 1% SDS, and sheared with a BioRuptor Sonicator (Diagenode, Denville, NJ, USA) for eight cycles of 5 min. ChIP was performed according to the manufacturer’s instructions (ChIP-IT High Sensitivity, cat# 53040, Active Motif, Carlsbad, CA, USA) using 10–30 μg of sonicated chromatin and the following antibodies for immunoprecipitation: H3K4 tri-methylation (Cat#04-745, Millipore, Burlington, MA, USA), H3K27 acetylation (Cat#39133, Active Motif), or H3K27 tri-methylation (Cat# 07-449, Millipore). Immune complexes were captured with 60 μl of 50% protein G sepharose beads, washed, and eluted in TE buffer. DNA fragments were purified using the ChIP-IT sensitivity kit filter columns. ChIP-seq libraries were prepared using KAPA DNA Library Preparation Kits for Illumina® (Cat# KK8234, Kapa Biosystems, Boston, MA, USA) and the library sequencing was performed on the HiSeq 4000 System (Illumina®).

Library construction and sequencing were performed by the Institute for Genomic Medicine (IGM) core research facility at UCSD. All histone modification ChIP-seq data were mapped to the UCSC mouse genome NCBI37/mm9 (July 2007). Bowtie 2 was used to map data to the genome with the following settings: the seed length was set as 33 with a maximum number of two mismatches allowed in the seed region, and reads aligning to multiple locations were discarded. After mapping, duplicate reads were removed using Samtools. ChIP-seq data from control islets are identical to the data set published previously as these samples served as controls for both BMP-2 stimulation (this study) and IL-1ß stimulation [69].

### Histone modification ChIP-seq analysis

Two replicates per condition were combined. The chromatin used for immunoprecipitation was likewise sequenced and used to normalize for differences in input. ChIP-seq analysis was performed using HOMER (Hypergeometric Optimization of Motif EnRichment). Peak calling. Peaks were required to have a twofold enrichment over the input sample (default setting by HOMER) and Poisson *P* value of <0.0000001. For H3K27ac and H3K4me3 “factor mode” was run. To account for the broader signal of H3K27me3 “–region –size 1000 –minDist 1000 –tagThreshold 50 mode” was run for peak calling. Differential Peak calling. Peaks that were differentially enriched between control and BMP-2 were identified using the getDifferentialPeaks command in HOMER. Peaks were required to have twofold enrichment between experimental groups (FDR Poisson *P* value of 0.0001). Gene annotation. Peaks were associated with the nearest gene, and the position of the TSS was determined using the annotatePeaks.pl command in HOMER, All parameters were left at their default setting. Motif Finding. Enriched motifs in ChIP-Seq peaks were identified using findMotifsGenome.pl using the -bg option to correct for background enrichment. The background was generated by removing enriched peaks from the full list of peaks in each sample using bedtools. All parameters were left at their default setting. Heat maps. Heat maps of differential peaks were generated using the annotatePeaks.pl function in HOMER. Tag densities within 50 bp bins spanning 6000 bp around the peak center were used to generate heatmap data. Rows were sorted decreasingly by the sum of the tag density in the center 2000 bp, and heat maps were generated using the heatmap.2 function of the gplots package in R. RNA-seq heat maps were also generated using the heatmap.2 function of the gplots package in R. If expression = =“up” & significant = =“yes”, the color is red; if expression = =“down” & significant = =”yes”, the color is green; others are blue. Genes with peaks overlapping selected for gene ontology analysis. Gene ontology analysis of genes with significantly differentially enriched ChIP-seq peaks was performed and visualized as described in the RNA-seq section. All genes with a detected peak overlapping a region 3000 bp upstream to 1000 bp downstream of their TSS were used as background and genes where the overlapping peak(s) were significantly differentially enriched, as defined above, were used as foreground, only terms containing between 10 and 500 genes with peaks were analyzed. The analysis was performed on genes with up- and down-regulated peaks separately.

### NeuroD1 ChIP-seq

About 1200 islets per condition (*N* = 2) were cross-linked for 10 min in 1.11% formaldehyde, lysed in ice-cold IP buffer (67 mM Tris-HCl (pH 8), 100 mM NaCl, 5 mM EDTA (pH 8.0), 0.2% NaN_3_, 0.33% SDS, 1,67% Triton X-100, 0.5 mM phenylmethylsulfonyl fluoride) and sonicated (Diagenode, BioRuptor) for 45 cycles of alternating 30 s intervals of sonication and rest. For each ChIP, chromatin corresponding to 1.4–6 μg of DNA was incubated overnight at 4 C° with NeuroD1 antibody (Cat#4373, Cell Signaling). Immune complexes were captured by incubation with protein G sepharose beads (GE Healthcare) for 4 h. The beads were washed three times with low-salt buffer (20 mM Tris-HCl (pH 8.0), 2 mM EDTA (pH 8.0), 1% Triton X-100, 0.1% SDS, and 150 mM NaCl) and one time with high-salt buffer (20 mM Tris-HCl (pH 8.0), 2 mM EDTA (pH 8.0), 1% Triton X-100, 0.1% SDS, and 500 mM NaCl). Chromatin was de-cross-linked in 120 μl 1% SDS and 0.1 M NaHCO3 for 6 h at 65 C°, and DNA fragments were purified using Qiagen MinElute PCR purification kit filter columns. Finally, ChIP-seq libraries were prepared using NEBNext® UltraTM DNA library Kit for Illumina® (E7370) and NEBNext® Multiplex Oligos for Illumina® (E7335/E7500). The PCR cycle number for each library amplification was optimized by running 10% of the library DNA in a real-time PCR reaction using Brilliant III Ultra-fast SYBR Green QPCR Master Mix (AH Diagnostic) and a C1000 Thermal cycler (Bio-Rad). DNA libraries were sequenced on a NextSeq 500 (Illumina®) by 38‐bp paired‐end sequencing.

NeuroD1 ChIP-seq data were mapped to the UCSC mouse genome NCBI37/mm10. Bowtie 2 was used to map data to the genome with the following settings: the seed length was set as 33 with a maximum number of two mismatches allowed in the seed region and reads aligning to multiple locations were discarded. After mapping, duplicate reads were removed using samtools.

### NeuroD1 ChIP-seq analysis

Two replicas per condition were combined. The chromatin used for immunoprecipitation was likewise sequenced and used to normalize for differences in input. ChIP-seq analysis was performed using HOMER (Hypergeometric Optimization of Motif EnRichment). Peaks were required to have twofold enrichment over the input sample (default setting by HOMER) and a Poisson *P* value of <0.0000001 and “-style factor” was run for peak calling.

Gene ontology analysis of NeuroD1 binding sites was performed using the Genomic Regions Enrichment of Annotations Tool (GREAT) v. 4.0.4 [74] on 2022-05-05 using default settings. All detected NeuroD1 binding sites were used as background and significantly differentially enriched binding sites, as defined above, were used as foreground. The analysis was run separately for sites with increased and decreased binding. A dotplot similar to the one generated for RNA-seq was manually constructed using the R package ggplot2 [75]. The top ten terms with an FDR less than 0.05 were visualized. On the x-axis is the percentage of genes that are within 1000 kb of at least one differentially enriched binding site out of all genes in the term that are within 1000 kb of at least one binding site, the size of the dot corresponds to the number of genes with one or more differentially enriched binding sites within 1000 kb. The color is mapped to –log10 (*P* value) as reported by GREAT.

### Statistical analysis

Results are given as mean (*N* < 10) or mean ± SD (*N* > 10). Comparison versus the respective control group was made by Student’s two-tailed paired *t*-test and a *P* value less than 0.05 was considered statistically significant. Statistics on RT-qPCR data were carried out using log-transformed data. For RNA and ChIP-seq correlation analysis, nonparametric Spearman correlation was used. Differentially expressed genes were identified with Cuffdiff using a statistical cutoff of FDR <0.05. ChIP-seq peaks exhibiting differential signals between treatment groups were identified using the getDifferentialPeaks command in HOMER (cutoff of twofold enrichment between groups and FDR Poisson *P* value of 0.0001).

## Supplementary information

Supplementary Figure 1-3

Original data files

aj-checklist

Supplementary datasheets 1-20

## Data Availability

Supplementary information is available at Cell Death and Disease’s website. Proteomics data are available via ProteomeXchange with the identifier PXD036655. For review; reviewer_pxd036655@ebi.ac.uk, Password: sjqtuZSk RNA-seq and ChIP-seq data are available at the GEO repository, GSE216233 with the reviewer token: ytalekagndirloz.
