# Supplementary material for: Beta cell dysfunction induced by bone morphogenetic protein (BMP)-2 is associated with histone modifications and decreased NeuroD1 chromatin binding
Source: Cell Death Dis. 2023 Jul 5;14(7):399. doi: 10.1038/s41419-023-05906-w (PMC10322916; doi:10.1038/s41419-023-05906-w)
Supplement: Supplementary file 1 — Supplementary Figure 1-3 [file 41419_2023_5906_MOESM1_ESM.pptx]

## Slide 1
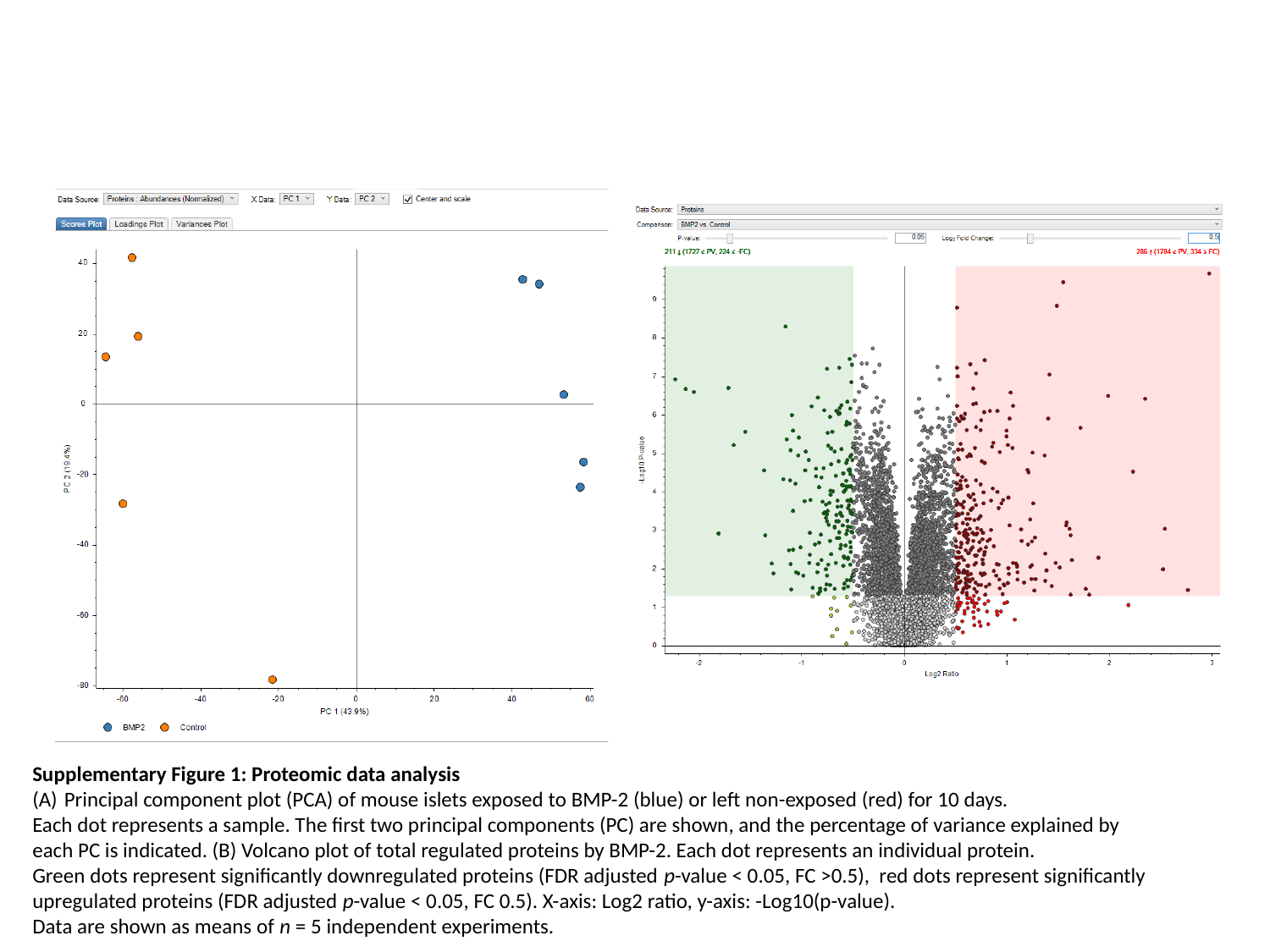

Supplementary Figure 1: Proteomic data analysis
Principal component plot (PCA) of mouse islets exposed to BMP-2 (blue) or left non-exposed (red) for 10 days.
Each dot represents a sample. The first two principal components (PC) are shown, and the percentage of variance explained by
each PC is indicated. (B) Volcano plot of total regulated proteins by BMP-2. Each dot represents an individual protein.
Green dots represent significantly downregulated proteins (FDR adjusted p-value < 0.05, FC >0.5), red dots represent significantly
upregulated proteins (FDR adjusted p-value < 0.05, FC 0.5). X-axis: Log2 ratio, y-axis: -Log10(p-value).
Data are shown as means of n = 5 independent experiments.

## Slide 2
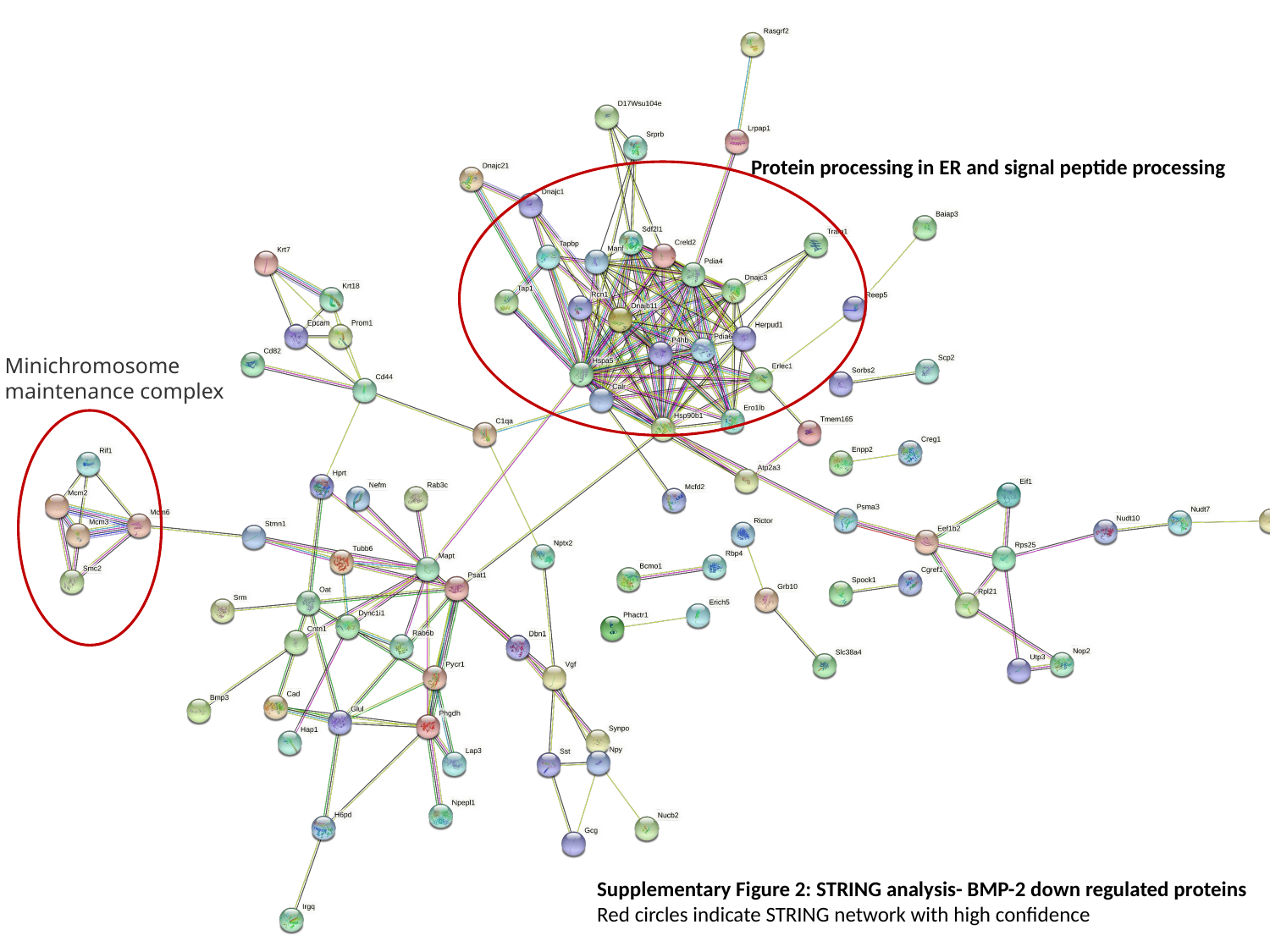

Protein processing in ER and signal peptide processing
Minichromosome
maintenance complex
Supplementary Figure 2: STRING analysis- BMP-2 down regulated proteins
Red circles indicate STRING network with high confidence

## Slide 3
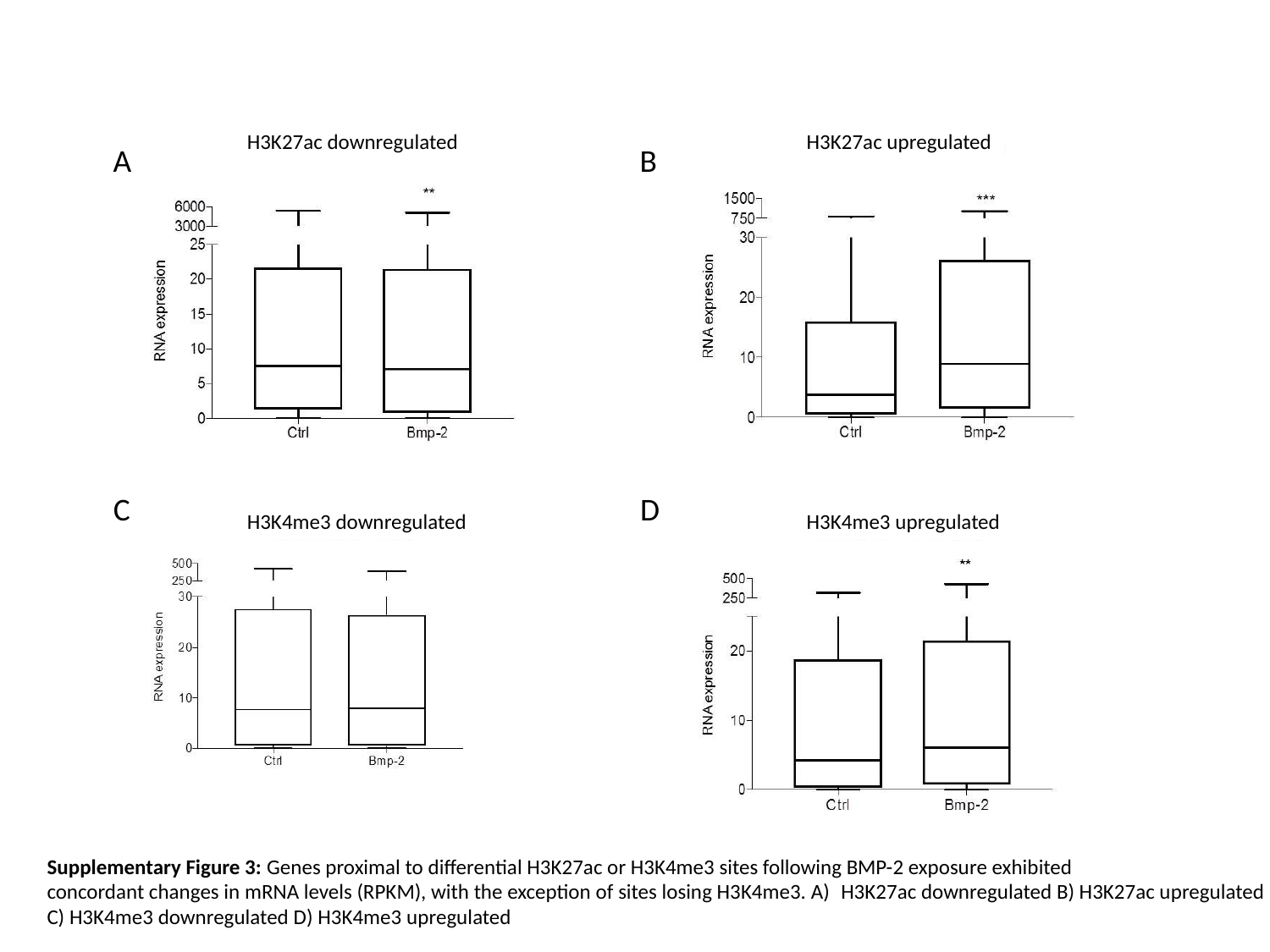

A
B
C
D
H3K27ac downregulated
H3K27ac upregulated
H3K4me3 downregulated
H3K4me3 upregulated
Supplementary Figure 3: Genes proximal to differential H3K27ac or H3K4me3 sites following BMP-2 exposure exhibited
concordant changes in mRNA levels (RPKM), with the exception of sites losing H3K4me3. A) H3K27ac downregulated B) H3K27ac upregulated
C) H3K4me3 downregulated D) H3K4me3 upregulated
