# Supplementary material for: Beta cell dysfunction induced by bone morphogenetic protein (BMP)-2 is associated with histone modifications and decreased NeuroD1 chromatin binding
Source: Cell Death Dis. 2023 Jul 5;14(7):399. doi: 10.1038/s41419-023-05906-w (PMC10322916; doi:10.1038/s41419-023-05906-w)
Supplement: Supplementary file 2 — Original data files [file 41419_2023_5906_MOESM2_ESM.pptx]

## Slide 1
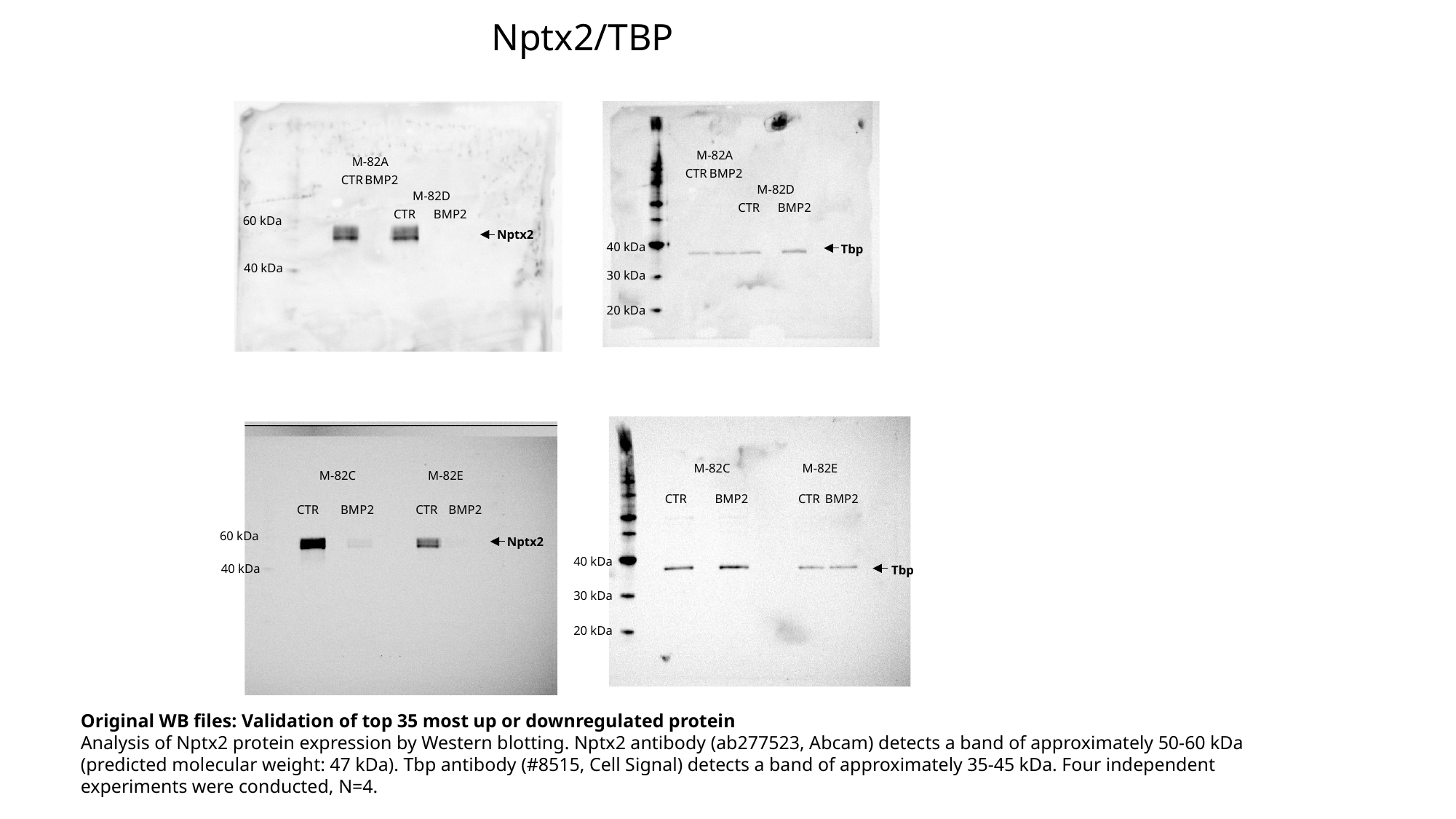

Nptx2/TBP
M-82A
M-82A
CTR
BMP2
CTR
BMP2
M-82D
M-82D
CTR
BMP2
CTR
BMP2
60 kDa
Nptx2
40 kDa
Tbp
40 kDa
30 kDa
20 kDa
M-82C
M-82E
M-82C
M-82E
CTR
BMP2
CTR
BMP2
CTR
BMP2
CTR
BMP2
60 kDa
Nptx2
40 kDa
40 kDa
Tbp
30 kDa
20 kDa
Original WB files: Validation of top 35 most up or downregulated protein
Analysis of Nptx2 protein expression by Western blotting. Nptx2 antibody (ab277523, Abcam) detects a band of approximately 50-60 kDa
(predicted molecular weight: 47 kDa). Tbp antibody (#8515, Cell Signal) detects a band of approximately 35-45 kDa. Four independent
experiments were conducted, N=4.

## Slide 2
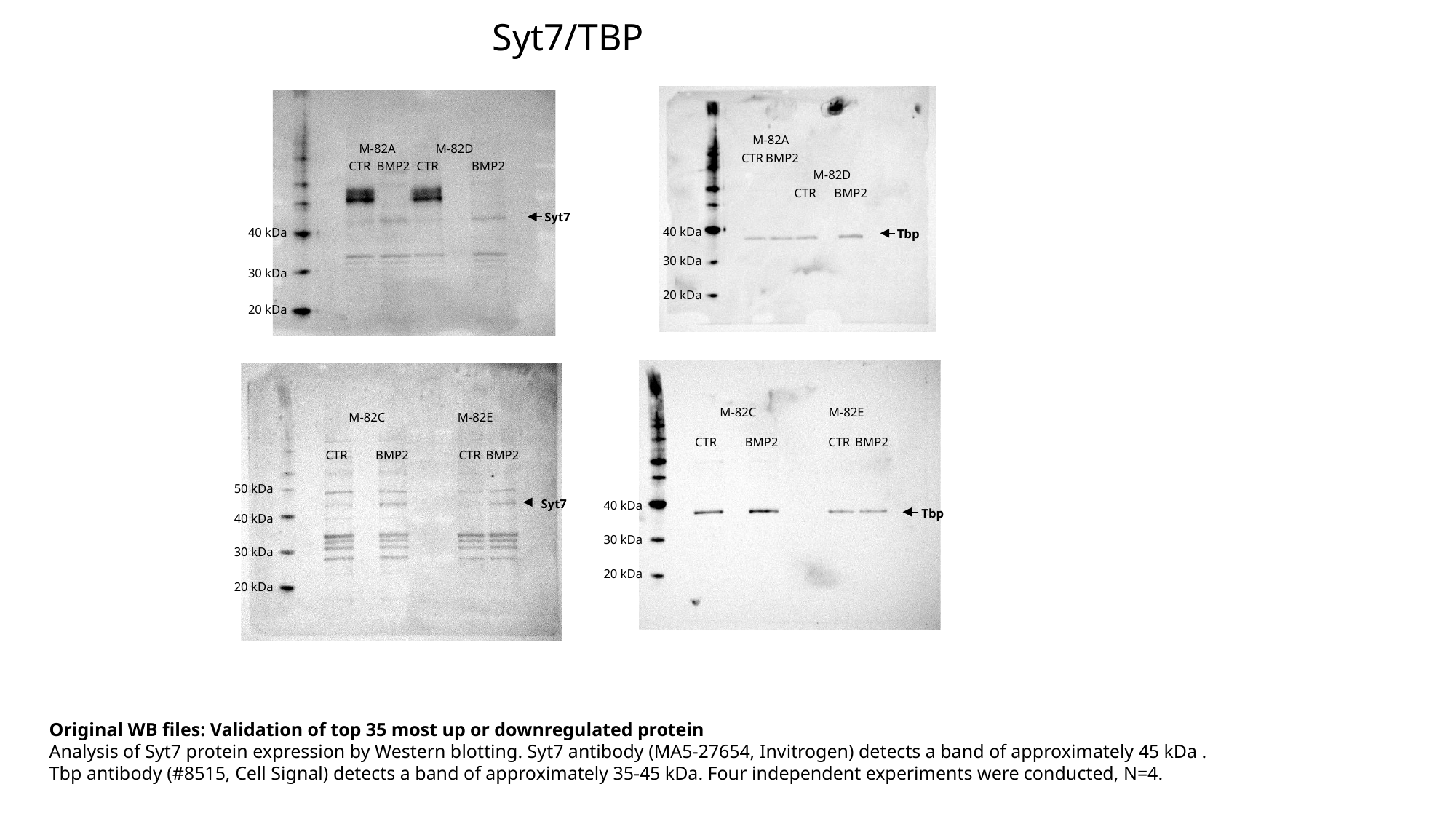

Syt7/TBP
M-82A
M-82A
M-82D
CTR
BMP2
CTR
BMP2
CTR
BMP2
M-82D
CTR
BMP2
Syt7
40 kDa
40 kDa
Tbp
30 kDa
30 kDa
20 kDa
20 kDa
M-82C
M-82E
M-82C
M-82E
CTR
BMP2
CTR
BMP2
CTR
BMP2
CTR
BMP2
50 kDa
Syt7
40 kDa
Tbp
40 kDa
30 kDa
30 kDa
20 kDa
20 kDa
Original WB files: Validation of top 35 most up or downregulated protein
Analysis of Syt7 protein expression by Western blotting. Syt7 antibody (MA5-27654, Invitrogen) detects a band of approximately 45 kDa .
Tbp antibody (#8515, Cell Signal) detects a band of approximately 35-45 kDa. Four independent experiments were conducted, N=4.
22/05/2023
2
